# Developing a translational triage research tool: part two—evaluating the tool through a Delphi study among experts

**DOI:** 10.1186/s13049-022-01035-z

**Published:** 2022-07-30

**Authors:** Amir Khorram-Manesh, Frederick M. Burkle, Johan Nordling, Krzysztof Goniewicz, Roberto Faccincani, Carl Magnusson, Bina Merzaai, Amila Ratnayake, Eric Carlström

**Affiliations:** 1 Institute of Clinical Sciences, Department of Surgery, Sahlgrenska Academy, Gothenburg University, 413 45 Gothenburg, Sweden; 2 Gothenburg Emergency Medicine Research Group (GEMREG), Sahlgrenska Academy, 413 45 Gothenburg, Sweden; 3 Institute of Health and Care Sciences, Sahlgrenska Academy, University of Gothenburg, 413 45 Gothenburg, Sweden; 4 Woodrow Wilson International Center for Scholars, Washington, DC USA; 5 Department of Aviation Security, Military University of Aviation, 08-521 Dęblin, Poland; 6 Emergency Department, Humanitas Mater Domini, 210 53 Castellanza, Italy; 7 Army Hospital, Narahenpita, Colombo, 08 Sri Lanka; 8 University of South-Eastern Norway, School of Business, 3616 Kongsberg, Norway

**Keywords:** Disasters, Mass casualty incident, Primary triage, Translational tool

## Abstract

**Background:**

There are different prehospital triage systems, but no consensus on what constitutes the optimal choice. This heterogeneity constitutes a threat in a mass casualty incident in which triage is used during multiagency collaboration to prioritize casualties according to the injuries’ severity. A previous study has confirmed the feasibility of using a Translational Triage Tool consisting of several steps which translate primary prehospital triage systems into one. This study aims to evaluate and verify the proposed algorithm using a panel of experts who in their careers have demonstrated proficiency in triage management through research, experience, education, and practice.

**Method:**

Several statements were obtained from earlier reports and were presented to the expert panel in two rounds of a Delphi study.

**Results:**

There was a consensus in all provided statements, and for the first time, the panel of experts also proposed the manageable number of critical victims per healthcare provider appropriate for proper triage management.

**Conclusion:**

The feasibility of the proposed algorithm was confirmed by experts with some minor modifications. The utility of the translational triage tool needs to be evaluated using authentic patient cards used in simulation exercises before being used in actual triage scenarios.

## Introduction

In mass casualty incidents (MCI), the number of casualties exceeds the locally available resources. Therefore, triage aims to prioritize casualties according to the severity of their injuries in a time- and resource-limited situation [1–8]. The concept of triage has evolved broadly during the last decades, resulting in primary, secondary, and tertiary triage, using the changes in patients’ vital signs over time [1, 6, 7, 9]. Primary triage allows sorting the victims, following a quick assessment of their condition, and performing life-saving interventions (LSI). Secondary triage aims to sort and refine victims’ conditions in a safer area with qualified medical capabilities and a longer observational time to achieve a more accurate diagnosis and course of action. In general, triage categories can be expressed descriptively (immediate; urgent; delayed; expectant), as a priority (1 to 4), or as color (Red, Yellow, Green, Blue), where the most critical category equals immediate, priority one or red color [1, 2, 6–8].

Four essential factors may affect hospital and prehospital triage systems: speed, precision, fairness, and compatibility [4, 9, 10]. However, prehospital systems allow for a more limited precision since speed frequently remains a priority, while hospital triage can often sacrifice time to maximize precision. Fairness implies an objective assessment of the patients according to vital parameters or mechanisms of injury, irrespective of age, gender, or any other individual aspect [1, 2]. Compatibility applies to translational triage systems across agencies and healthcare [1, 2, 9, 11, 12]. Triage systems may over-triage or under-triage a patient. Generally, over-triage is acceptable in prehospital/emergency settings (up to 25–30%), emphasizing the risk of faulty categorization in a speedy process. While under-triage should be avoided, it requires more time to establish accurate diagnoses, and thus, might increase the mortality rate in a time-limited situation [2, 8, 10–14].

Different triage systems have been designed for organizational, situational, regional, and national use, and have been presented as crude algorithms, flowcharts, and complex scoring systems [6–8, 10, 15]. This heterogeneity constitutes a threat in the event of an MCI, which often involves multinational and multiagency rescue teams with diverse educational and cultural backgrounds [2, 16, 17]. There have been several attempts to achieve a global or even national consensus without any results, partly due to a lack of actual research behind the origin or refinements of the various systems and the anecdotal nature of the evidence of a system’s efficacy [11, 17–20].

**Fig. 1:**
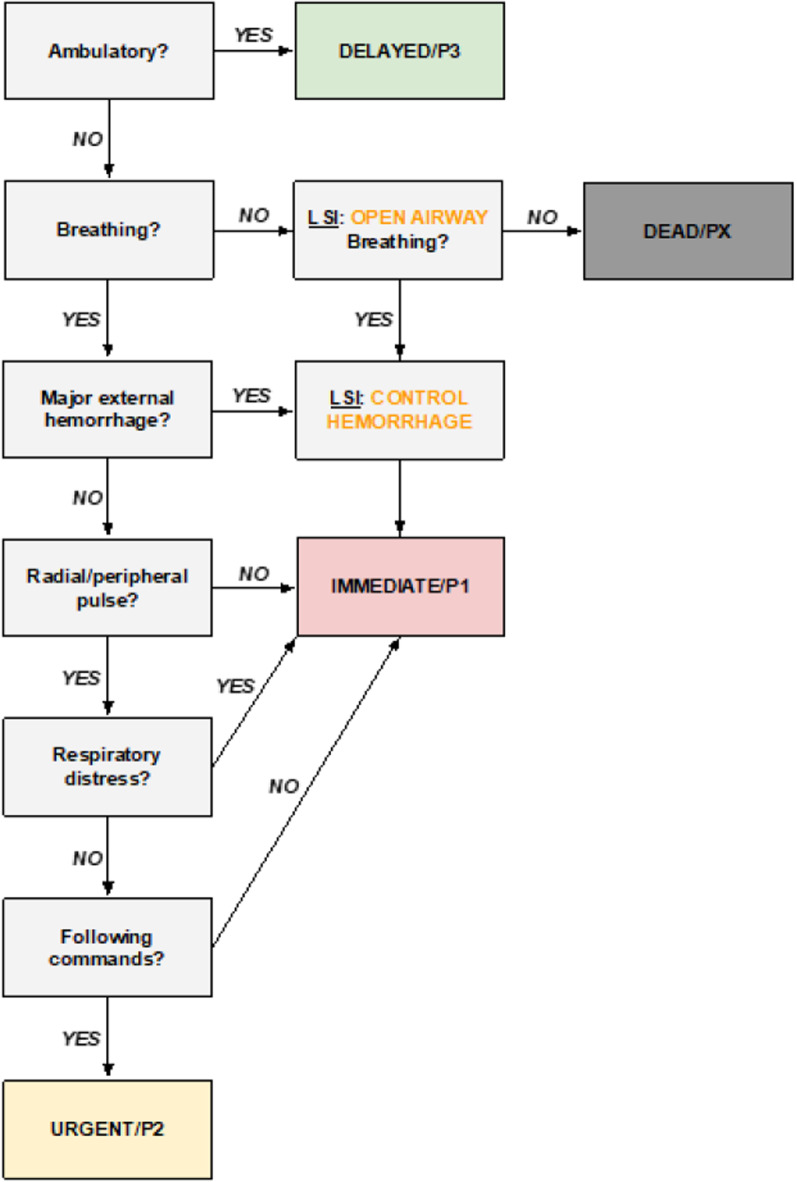
System constructed from majority criteria, modified according to discussion regarding criteria and lifesaving interventions (LSIs)

In a recently published paper, the authors described the first step in a multistep procedure to evaluate the possibility of creating a translational triage tool (TTT) for prehospital use in MCI [4]. Using a Rapid Evidence Review, consisting of a systematic literature review, the outcomes were merged and analyzed through content analysis, and a universal system approach was developed. They suggested a change of paradigm from the current number-based prehospital triage to a symptom-based (physiological) triage [4], emphasizing that the power of triage lies in the ability of providers to recognize and decode vital signs and clinical symptoms [1]. The paper presented a combined criteria system to be considered to display the results of an assessment, avoiding yet another triage system (Fig. 1). Discussing several steps in the algorithm, the paper suggested that a simplified tool could translate diverse primary triage into one to enhance the compatibility of all triage systems and ensure patient safety [1, 4]. Further research was recommended to verify the decisive stages proposed in their algorithm.

The proposed translational triage tool (Fig. 1) emphasized the following steps:

1. *Ambulation:* As the first divider in a primary triage [8], a walking casualty can receive and elicit a motor response to the command to walk to a secure rendezvous point, indicating intact brain and circulatory functions and no major structural damages [6, 9, 10, 15].
2. *Breathing/open airway:* As a deciding factor for categorizing casualties as dead [8], lack of spontaneous breathing generally implies delayed resuscitation in a resource-limited environment and allowing 1–2 attempts of a lifesaving intervention [LSI] [1, 6, 21].
3. *Respiratory rate:* Instrumental for finding preventable deaths in casualties with primary injuries affecting the airways/lungs [21, 22], it demands an interval of acceptable values when counting in a conceivably loud, stressful, and weather-affected surrounding [4, 9, 15]. It may also fail to give a fair picture of the casualties’ actual condition due to its dynamic nature and association with the patient’s psychological condition (psychological shock and psychogenic hyperventilation) [15, 22–26], favoring a quick assessment of victims’ respiratory distress, which may require some level of basic medical training and intervention [18, 20].
4. *Radial/peripheral pulse:* An estimation of blood pressure to identify a hypotensive trauma casualty with a high risk of life-threatening external or internal bleeding is supported by numerous studies [27, 28]. However, using a sphygmomanometer in the resource-limited and stressful event of an MCI is inconvincible [25, 26, 29]. Thus, the palpation of the radial pulse will be rational in prehospital triage systems to prioritize a casualty *without* a palpable radial pulse higher than one with a palpable pulse or to differentiate the casualties with a high probability of internal bleeding from the ones that need an intervention in the field [18, 20]. The algorithm proposed a question before the palpation of the pulse; is there any “*Major external bleeding: YES/NO?*” If YES, proceed with an LSI, and if: NO, proceed with “*Radial pulse palpable: YES/NO?*”
5. *Following commands:* Whether a casualty can follow commands or not indicates if a victim can receive and process auditory information and turn it into a verbal or motor response, i.e., an intact or minorly injured CNS and circulation [8]. This assessment of verbal or motor response to stimuli is a part of the Glasgow Coma Scale (GCS) [30], which evaluates the level of consciousness in patients with acute brain injury. The motor component of the scale is a predictor of both the need for an LSI [31] and the risk of dying [32, 33] in studied groups of trauma patients.
6. *Lifesaving interventions (LSI):* The performance of LSI depends on (1) who will perform the triage and (2) what resources/equipment are available in the field? A triage system in its simplest form should be usable for anyone, but that would require a redundancy level that might not provide correct prioritization from a strictly medical point of view. Correspondingly, a system so uncomplicated that *anyone* could use it leaves no room for LSI in the field, e.g., controlling major bleeding by applying a tourniquet [34, 35]. Secondly, the MCI triage system must be constructed, taking austere conditions and minimal equipment requirements into consideration. Major bleeding has been identified as the primary determining factor in preventable trauma death which warrants at least an attempt at controlling the bleeding on the prehospital scene. This could be accomplished with minimal equipment; makeshift tourniquets can be made from clothing, and direct pressure can be applied by bystanders, another victim, or, in some cases, even the injured casualty [34].

This study aimed to evaluate and verify the proposed steps in the previous report, which was based on a systematic review, by using a panel of experts in triage through a Delphi study.

## Method

### Study design

Through a Delphi study, the opinions of experts with documented knowledge and experience in triage were searched by assessing the extent of agreement or disagreement on a certain statement. The method is a reliable forecasting tool that helps develop new concepts, sets the direction of future research, and has been used in multiple studies to establish consensus in several subject areas [36–39]. Emphasizing the anonymity and confidentiality of the method, no demographic data, such as age or gender, were collected since the core requirement for participation in this study was the participants’ specialty and experience in managing emergencies and disasters at the hospital as well as prehospital settings. This requirement was met since experienced professionals who did not participate in the study themselves recommended all included participants. The Delphi process consists of several rounds. The group’s collective response results, i.e., percentage agreement/disagreement to each statement, are collected in the first round. Undecided responses are excluded depending on the options used. If the consensus is not achieved, the participants are asked to reconsider their responses considering the entire group’s responses. Round two includes the previous round’s statement with no achieved consensus and new statements derived from the free-text responses to round one. This aims to clarify decisions that could then be added as a new statement in round two [36–41].

In this study, a developed survey that included statements created based on the previously published algorithm for primary triage [4] was ranked by the participants using a 5-point Likert scale. The options used were’completely agree,’ ‘agree,’ ‘undecided,’ ‘disagree,’ and ‘completely disagree.’ A free-text response was available for each statement in this round, providing the opportunity to elaborate or explain responses. All responses were evaluated carefully and matched to the comments given. Statements with no consensus were presented again in round two, along with new statements derived from the free-text responses from round one. A third survey was planned if consensus was not achieved, and the evaluation of the results showed a possibility of reaching a consensus on any of the statements [36, 37, 39, 41, 42]. All surveys were administered using Google Forms, and survey links were distributed through email.

### Survey development

Two of the study team’s experts (AK, EC) developed the statements for the survey based on the review yielding the published algorithm and critical reviews of two independent reviewers [4]. To meet the study objectives, critical steps in the algorithm and all points mentioned by the independent reviewers were included. This process resulted in 13 statements analyzed independently and critically by seven other authors (AR, BM, CM, FB, JN, KG, RF). All statements were edited and refined. The new statements in round two were developed from the comments in round one and were sent out after being evaluated by the research team members. The survey was piloted with five academics with trauma and emergency medicine-related experience, including people with experience in major incidents, wars, and current pandemics. Further feedback was received to improve the structure, readability, and comprehensiveness of statements and determine whether additional statements were needed. The entire process followed the method described by earlier studies [36–41].

### Expert panel recruitment

A non-probability purposive sample of thirty recommended participants, trauma, and emergency medicine specialists, was invited via email to participate in this Delphi study. Sampling was purposive to ensure that invited participants met the inclusion criteria (Table 1).

**Table 1:** Expert panel members

No	Specialty (Physician/Nurse)	Current position/Teaching & Research	Country
1	Emergency Medicine (P)	Consultant Emergency Department/Yes	Sweden
2	Surgery (P)	Consultant trauma/Instructor/Yes	Sweden
3	Emergency Medicine (P)	Consultant Emergency Department/Yes	Thailand
4	Emergency Medicine (P)	Consultant Emergency Department/Yes	Thailand
5	Emergency Medicine (P)	Consultant Emergency Department/Yes	Thailand
6	Emergency Medicine (P)	Consultant Emergency Department/Yes	Thailand
7	Emergency Medicine (P)	Emergency Department/Yes	Iran
8	Emergency Medicine (N)	Emergency Department/Yes	Italy
9	Anesthesiology (P)	Intensive care/Yes	Italy
10	Emergency Medicine (N)	Prehospital Consult/Yes	UK
11	Emergency Medicine (P)	Consultant/Educator//Yes	UK
12	Surgery (P)	Consultant Surgery, trauma/Yes	UK
13	Emergency Medicine (N)	Prehospital/Emergency/Yes	Australia
14	Public Health and Emergency (P)	Consultant/Yes	New Zealand
15	Emergency Medicine (N)	Prehospital/Yes	USA
16	Emergency Medicine (P)	Consultant Emergency department/Yes	Belgium
17	Anesthesiology (P)	Consultant/Yes	Norway
18	Emergency Medicine (P)	Consultant/Yes	Poland
19	Surgery (P)	Consultant/Yes	Netherland

A minimum of 12 respondents is generally considered sufficient in Delphi studies to verify an achieved consensus and avoid decreasing returns, influencing the validity of the results associated with larger sample sizes [39, 40]. Table 1 shows the characteristics of the recruited expert panelists.

### Inclusion criteria

All participants were required to be active, experienced (more than/equal to 10 years) medical staff (nurses or physicians) in the fields of emergency and or disaster medicine and affiliated with an academic institution. To complete the Delphi process, participants were required to respond to all rounds. As reported by previous Delphi studies, a dropout rate of 20% was expected over the three rounds [41].

### Ethics

The study complied with Swedish law's ethical guidelines and principles and was exempted from ethical approval requirements. In Sweden, where the study was conducted, ethical approval is mandatory if the research includes sensitive data on the participants such as race, ethnical heritage, political views, religion, sexual habits, and health or physical interventions or employs a method that aims to affect the person physically or psychologically [42]. All participants freely volunteered to partake and were assured they could withdraw without penalty. They received information including the study’s design, purpose, absolute confidentiality, anonymity, and secure data storage. Written information was provided before each round digitally and before participants were asked to choose their options.

### Data analysis

Descriptive statistics were used. The consensus was defined as > 70% of participants agreeing/completely agreeing or disagreeing/completely disagreeing with a statement. This level of agreement has been considered appropriate in previous Delphi studies [38, 43]. All ‘undecided’ responses were matched before excluding the group response to ensure that the reported percentage agreement or disagreement for each statement represented the consensus among only those who felt they knew the answer.

## Results

### Round one

The first round started in the second half of January 2022. An email was sent to all 30 selected experts explaining the study’s purpose and methods. They were asked to declare whether they would like to partake in the study or not. A sum of 19 participants accepted to participate (63.3%). Participants originated from all parts of the world, but representatives from African countries and South America did not reply. The link for the google form containing all 13 statements was sent to 19 participants who were asked to express their opinions within a week. Reminders were sent if no answers were received. All 19 participants replied to the first round (100%). All responses were evaluated carefully and matched to the comments given. Statements were grouped in a) completely agreed/Agreed, b) undecided, and c) completely disagree/Disagree. According to the guidelines for the Delphi study, undecided responses were carefully evaluated before exclusion (see limitations) [36, 41, 43]. Normally, the statements with no consensus would be presented again in the second round, with new statements derived from the free-text responses from round one.

**Fig. 2:**
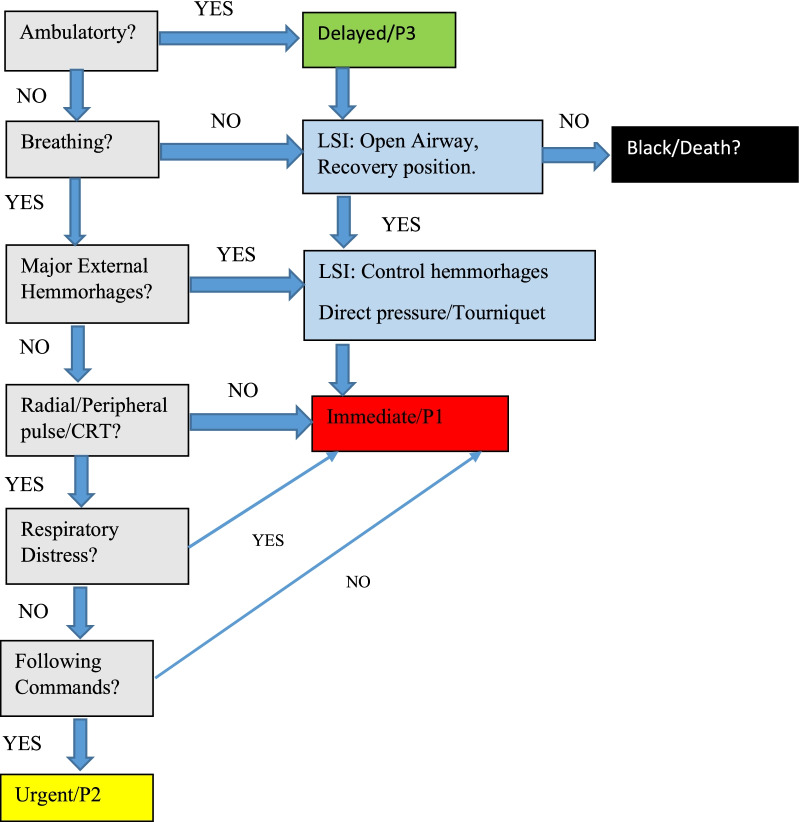
The modified algorithm constructed from experts’ opinions

All statements in the first round passed the 70% limit for consensus, and unexpectedly, there was a consensus for each statement, as shown in Table 2. Over 80% of the participants agreed on statements 1,3,4,5,6,8,9,10,11,12,13. There was a 75% consensus on statement 2. However, many of those who disagreed recommended the statement given in the next step, the third statement (answering the actual statement and comment was mandatory before continuing to the next). There was a disagreement on statement 7, which initially was calculated as 60% completely disagree/disagree and could motivate a new round of discussion. However, some of the comments from the completely agreed/agreed section were conditional, such as “First chin lift, head tilt or jaw thrust should be implemented.” These responses were moved to the opposing column; thus, a 70% disagreement was achieved, and consequently none of the statements needed to be transferred to the second round. Figure 2 shows the modified algorithm which was constructed from experts’ opinions. It is similar to the one previously published but modified based on the discussion highlighted in this study.

**Table 2:** Statements and responses in the first round of the Delphi study. Response 100% (19/19)

Statements	Completely agree/agree %	Completely disagree/Disagree %	Some Comments, given by participants
1. All ambulatory cases, irrespective of symptoms, will be PRIMARY triaged as Green (delayed/P3)	81	19	Ambulatory circumferential burn/ severe oral injury? Observe burn inhalation
2. Absence of breathing is enough to distinguish between salvageable or dead (PX) victims	75	25	Only after clearing the airway Should declare the patient as "no sign of life" since death is a legal definition?
3. In question no. 2, the lack of breathing is enough to initiate intervention, such as positioning of the airway (Jaw trust/chin lift/head tilt). Please note no medical devices (e.g., Guedel) are available	89	11	The lack of staff can make maneuvers such as "chin lift" or "jaw thrust" useless. The lateral safety position might be useful despite the potential risk of spinal injury If no breathing, just move on. Spending more time with victims to open airways may delay treatment of others
4. One intervention attempt is enough to validate between salvageable/dead (PX)	80	20	It allows managing multiple patients The opening airway is enough to determine to breathe
5. Observation of major external hemorrhage is enough to triage the victim Red/P1 patient	95	5	This is in line with the C-ABC philosophy A simple sign to detect and indicates a life-threatening situation Immediate action to control
6. In question no. 5, the external hemorrhage is enough to initiate intervention	100	0	Needs to be fast and efficiently such as tourniquet and/direct pressure That is according to the C-ABC concept Interventions always depend on the presence of sufficient staff Staff must cut clothing and identify the site of bleeding/ P1
7. When initiating intervention according to question number 6, applying direct pressure to active bleeding in the thorax/abdomen is a sufficient intervention	70	30	A tourniquet should be used as the last resort Direct pressure is the most feasible intervention in proximal non-compressible injury in a major incident setting
8. When initiating intervention according to question number 6, applying a tourniquet to extremities, above active bleeding, if the direct pressure fails or you have to release it, is a sufficient intervention	94	6	This approach is effective in stopping bleeding and takes less time A tourniquet is more sufficient than direct pressure alone Direct pressure OR tourniquet—pick one and move one
9. With no external hemorrhages, it is sufficient to evaluate the victim’s circulatory status by palpating radial or peripheral pulse	85	15	And Capillary Refill Carotid
10. The lack of radial or peripheral pulse is enough to triage the victims as Red/P1	81	19	Capillary refill more than 2 S Check quickly for any other indicators, Carotid or capillary refill
11. Victims, who breathe, have radial or peripheral pulse but show signs of respiratory distress, i.e., having trouble breathing or not getting enough oxygen (a bluish color seen around the mouth, on the inside of the lips, or the fingernails may happen) will be triaged as Red/P1	95	5	These signs indicate critical status Signs or symptoms of abnormal breathing should be checked and treated asap Airway, breathing, circulation. Any breathing problems should be dealt with as a priority until a secondary survey is done
12. Victims, who breathe, have radial or peripheral pulse, and have no respiratory distress, who are unable to follow commands, are triaged as Red/P1	88	12	A reduced level of consciousness may be an indicator of severe head injury and/or hypovolemia Severe traumatic brain injury might be present
13. Victims, who breathe, have radial or peripheral pulse, and no respiratory distress, who are able to follow commands, are triaged as Yellow/P2	95	5	This warrants that they have some kind of injury Immediate intervention or procedure is not indicated at that time Stable enough to wait a bit until P1's are treated

Reading all comments carefully, the research team recognized that despite defining primary triage in the introduction of the survey, some participants had difficulties comprehending the definition. Several participants asked for interventions, which were not possible to do during primary triage. There was also a doubt about what was the most important factor in primary triage, the time, or an accurate diagnosis? Finally, there seemed to be different opinions on how many victims can be managed during a very constrained situation with limited time as can occur during a mass casualty incident. The comments from participants led to new statements for the second round (Table 3).

**Table 3:** Statements and responses in the second round of Delphi study: response rate 100% (19/19)

Statements	Completely agree/Agree %	Completely disagree/Disagree %	Other
1. Healthcare providers/First responders conduct primary triage right at the incident scene to assess life-threatening injuries efficiently and to make life-saving interventions quickly, in a time- and resource-limited environment, which does not allow all victims to be treated immediately. Since both healthcare providers and victims may face danger and hazards, there is neither time for detailed investigation, nor treatment	100	0	
2. Time is the most significant factor in primary triage	100	0	
3. Diagnose accuracy is the most significant factor in primary triage	29	71	
4. Mass Casualty Incident (MCI) can be defined as “an overwhelming event, which generates more patients at a time than locally available resources can manage using routine procedures [43]. This definition is ambiguous and very general. To put yourself in a situation, one should realize how many victims one healthcare provider could manage during an MCI when both time and resources are limited. In the most favorable condition (weather, space, ordinary staff) in a mass casualty incident (i.e., when resources and workforce are insufficient), one healthcare provider could use a primary triage algorithm to manage xx victims simultaneously without having any other responsibility			> 2 patients

### Round two

The second round started in the first half of February 2022. An email was sent to all 19 experts who responded to the survey in the first round. The outcomes of the first round were shortly described, and the reason for creating the new statement for the second round was explained. Reminders were sent if no answers were received initially.

All 19 participants replied to the second round (100%). All responses were evaluated as previously explained. Table 3 shows the statements and responses in the second round of this Delphi study. Four participants were unsure about the first statement and the definition of “primary triage.” The remaining 15 participants agreed on the definition, and no one disagreed.

Although three participants were unsure about the second statement, the rest (n = 16) agreed that time is the most significant factor in primary triage and management of the victims. Five participants were undecided whether diagnosis accuracy is needed in primary triage, and ten disagreed. Of the remaining four participants, only one completely agreed that diagnostic accuracy is a significant factor in primary triage. The last statement in the second round proved to be a difficult question, but 42% reported that a sole healthcare provider could only manage two victims simultaneously (assessing and performing simple and vital life-saving measures). Around 32% chose three victims, 11% chose 4, and 15% chose five or more victims.

## Discussion

This study evaluated critical steps in a previously published translational triage research tool (TTT) [4], which attempted to standardize prehospital triage decision-making in MCIs. Undertaking a Delphi study group consisting of several trauma and emergency medicine experts, the study’s outcomes confirm the feasibility of the previous algorithm with minimal necessary revisions which primarily aim to clarify interventions that need to be implemented in some decisive and critical steps. This study also succeeded in achieving a consensus on the definition of mass casualty and primary triage, along with a suggestion on when an algorithm in an MCI should be used by defining the number of victims per one healthcare provider.

Having experienced the current pandemic, many of our 19 experts had experienced the difficult task of prioritizing patients in a constrained and severe, slow-onset major incident. The number of participants in this study, assessing a sudden-onset major incident, was sufficient to conduct a meaningful Delphi study. The limit for consensus on agreement or disagreement was higher (over 80%) for most of the statements (standard 70%), indicating a broad level of agreement among study participants [36–41, 43].

The most challenging part of this study was to make the statements understandable. Since the panel consisted of international participants with diverse native languages, this challenge was met by engaging a multinational study group, including native English speakers, to test the feasibility and comprehensiveness of the questions. Despite this effort and issuing necessary definitions, some comments indicated a diverse understanding of both an MCI and primary triage. These topics were managed later in the second round of the study to achieve a consensus on both definitions. There were some novel and critical steps in the previously published algorithm, the first of which was to achieve a consensus on the first dividing factor in an MCI triage system. Walking victims are triaged as P3/Green in most triage systems. This was confirmed by 81% of participants. A few participants expressed concern about burn patients with circumferential or possible inhalation injury. Although the comments are valid, in an MCI with limited time and resources, walking patients should be directed to the secondary triage area for further assessment [6, 9–13, 15, 44]. The secondary triage should be performed at a secure site close enough not to expose victims to unnecessary danger.

Although 75% of participants agreed with the second statement, the main concerns remained on how to clarify someone was dead after ensuring that the victim has an open airway since confirmation of death is a legal definition [45]. This catalyzed the first modification in the algorithm, the terminology used “dead/PX, which also concerned statements 3 and 4. First, dead/PX should be substituted with a “Black” triage tag to allow the death to be confirmed by a physician, as it is legally recommended in several countries [46]. Furthermore, although 89% of the participants agreed on one attempt to save a victim with absent breathing, most participants (80%) recommended only simple maneuvers such as opening, clearing, and repositioning the airway. Jaw thrust and chin lift were not recommended since these measures are staff-dependent, and the lack of staff in an MCI can make them useless [47].

Statements 5 to 10 dealt with major external hemorrhage and their assessment and management. Observing external hemorrhages should prompt immediate action since it might quickly lead to hypovolemia and death [27–31]. This was confirmed by 95% of the participants. All participants (100%) also agreed that such an observation is enough to prioritize the victim as P1 and initiate intervention. However, only 70% believed direct pressure should be used to manage such bleeding. Some recommended abdominal tourniquets, which are controversial, time-consuming, and not carried by many healthcare providers during a primary triage assessment, although victims’ clothes might be used for such a maneuver [48, 49]. To the best of our knowledge, direct pressure is the most feasible intervention in proximal non-compressible injury in a major incident setting [49]. Tourniquets on extremities have also been debated for years, with several experts recommending their use while others do not. Ninety-five percent of experts in this study recommended using a tourniquet on the bleeding extremities. They confirmed that the approach is more effective than direct pressure on extremities in stopping bleeding and takes less time [50, 51]. One expert stated, “*Direct pressure OR tourniquet—pick one and move on*” emphasizing the significance of time in MCI management. Another point of discussion was the assessment of the circulatory status of the victim by using radial or peripheral pulses. While 85% agree with what is indicated by the algorithm (the use of radial or peripheral (carotid) pulse), several participants recommend capillary refill as a second option. Over 81% of the participants also agree with the TTT algorithm that the lack of radial and peripheral pulses can be used to triage the victims as Red. However, several participants asked for extra options before making a final decision of prioritizing by citing the limited resources and staff as the main reason for the optimization of the measures. In some cases, pulses might be hard to obtain; the same applies to capillary refill in cold environments [52, 53]. However, both options should probably be available and included in the algorithm. Thus, the second modification in the algorithm.

The eleventh statement concerned respiration and respiratory rate. The statement aims at substituting number-based criteria with symptom-based criteria. About 91% of participants agreed that respiratory distress, a qualitative measure, can be used to triage a victim as P1 [1, 54]. Lastly, the last two statements aim at the mental assessment/condition of a victim with no sign of respiratory or circulatory insults or injuries. Following or not following commands will triage a victim as P1 or P2. From 88 to 95% agreed with the statements, although some would like to down-triage the victims as soon as possible [55, 56].

In summary, round one of this Delphi study was unexpectedly positive and a consensus was reached in all statements. However, the comments given in the first round indicated some misunderstanding of what constitutes an MCI and primary triage. Therefore, the second round was initiated with four new questions and a final free-text alternative to express other thoughts. Consequently, 100% agreed on the following definition for primary triage.

Healthcare providers/First responders conduct primary triage right at the incident scene to assess life-threatening injuries efficiently and make life-saving interventions quickly, in a time- and resource-limited environment, which does not allow all victims to be treated immediately. Since both healthcare providers and victims may face danger and hazards, there is neither time for detailed investigation nor treatment.

Additionally, 100% agreed that time is the most significant factor in primary triage. However, despite agreeing with the definition of primary triage and the significance of time, 29% still believe that the victim should have an accurate diagnosis during the primary triage. How this can be possible during a time and the resource-limited situation needs to be further investigated, probably through face-to-face interviews. Finally, to calculate the number of manageable victims per healthcare provider, the participants were given the final question: how many victims one healthcare provider could manage during an MCI when both time and resources are limited?

In the most favorable condition (weather, space, ordinary staff) in a mass casualty incident (i.e., when resources and workforce are insufficient), one healthcare provider could use a primary triage algorithm to manage xx victims simultaneously without having any other responsibility.

Around 42% chose two victims per healthcare provider, while 33% chose three victims per healthcare provider. The outcome is clearer when taking the MCI situation and definition into consideration. As far as we understand, the number of manageable victims per healthcare provider in an MCI is not considered in previously suggested triage models. Such consideration is particularly important in critical circumstances with a need for decision-making, given the ambiguities in defining disasters, and when to use a triage model tailored for such events [1, 4, 57, 58].

## Limitation

Although 4-point scales may produce stable findings [41], an undecided option in this study demonstrates the uncertainty in emergencies and in facing real consequences. In some studies, this option is replaced by ‘don’t know’ to emphasize that some participants may not know how to answer certain statements. Another limitation might be the number of Delphi rounds. However, appropriate Delphi surveys are built upon an iterative process and controlled feedback to generate consensus. The closing criteria in most of the Delphi studies include consensus achieved after usually two rounds [59]. In addition, other reports have shown that there was a further shift in opinion towards the group opinion if the response rate in round 2 was more than 75% (i.e., the category then yielded even greater consensus) [60].

## Conclusion

The outcome of this study confirms the feasibility of using a translational triage tool (TTT) with some minor modifications. Having achieved this result, the tool needs to be used in situations where the outcomes of the diverse triage model can be compared. The next step is to assess this tool by using authentic patient cards used in simulation exercises. “Medical Response to Major Incidents” is a scenario-based simulations exercise which uses authentic patient cards based on real patient data from real incidents, such as the Madrid and London Bombings [56]. The data and the patient cards can be used to examine the utility of the proposed Translational Triage Tool.

## Data Availability

All data generated or analyzed during this study are included in this published article or supplementary files. References used in the literature review are available on the world web.

## Abbreviations

MCI: Mass casualty incidents
P: Priority
CNC: Central Nervous System
LSI: Life saving intervention
RR: Respiratory rate
